# Supplementary material for: What does it take to learn the rules of RNA base pairing? A lot less than you may think
Source: bioRxiv. 2025 Aug 2:2025.07.31.668042. Preprint. [Version 1] doi: 10.1101/2025.07.31.668042 (PMC12324431; doi:10.1101/2025.07.31.668042)
Supplement: Supplement 1 [file media-1.gz › supplemental_material/src/rscape_v2.6.0.tar.gz!/rscape_v2.6.0/lib/infernal/Manuscripts/banded-cyk/tbl-rmarkmerlist.doc]

|  |  | entropy |  | summary | family-specific |
| --- | --- | --- | --- | --- | --- |
| program | prior | (bits) | β | MER | MER |
| blastn | - | - | - | 216 | 188 |
| infernal 0.55 | plus-1 | - | - | 232 | 180 |
| infernal 0.72 | plus-1 | - | - | 215 | 187 |
| infernal 0.72 | plus-1 | 1.46 | - | 208 | 191 |
| infernal 0.72 | informative | - | - | 177 | 158 |
| infernal 0.72 | informative | 1.46 | - | 105 | 90 |
| infernal 0.72 | informative | 1.46 |  | 114 | 96 |

Table 6: **Rfam benchmark MER summary statistics.** “prior”: “plus-1” if uninformative Laplace plus-1 priors were used; “informative” if new Dirichlet priors were used. “entropy”: target model entropy in bits for entropy weighting; “-” if entropy weighting was not used. “β”: tail probability loss for banded calculation used; “-” if search was non-banded. “summary MER”: MER across 51 benchmark families; “family-specific MER”: MER for each family, summed over all 51 families. Program versions: Row 1: WU-BLASTN-2.0MP –kap -W=7. For infernal version 0.55, window length values (*W*) were preset as calculated in version 0.72 with plus-1 priors.
